# Touch Locating and Stretch Sensing Studies of Conductive Hydrogels with Applications to Soft Robots

**DOI:** 10.3390/s18020569

**Published:** 2018-02-13

**Authors:** Yanmin Zhou, Bin He, Zhe Yan, Yinghui Shang, Qigang Wang, Zhipeng Wang

**Affiliations:** 1 School of Electronics and Information Engineering, Tongji University, Shanghai 201804, China; yanmin.zhou@tongji.edu.cn (Y.Z.); 1631527@tongji.edu.cn (Z.Y.); wangzhipeng@tongji.edu.cn (Z.W.); 2 School of Chemical Science and Engineering, Tongji University, Shanghai 200092, China; shangdiyinghui@163.com (Y.S.); wangqg66@tongji.edu.cn (Q.W.)

**Keywords:** intelligent material, soft robots, sensing, 3D printing, driving–sensing integration

## Abstract

Soft robots possess great potential in environmental adaptations, while their environmental sensing abilities are critical. Conductive hydrogels have been suggested to possess sensing abilities. However, their application in soft robots is lacking. In this work, we fabricated a soft and stretchable gel material, introduced its sensing mechanisms, and developed a measurement setup. Both experimental and simulation studies indicate strong nonlinearity of touch locating on a square touch panel with Cartesian coordinates. To simplify the touch locating, we proposed a touch locating system based on round touch panels with polar coordinates. Mathematical calculations and finite element method (FEM) simulations showed that in this system the locating of a touch point was only determined by its polar radius. This was verified by experimental studies. As a resistor, a gel strip’s resistance increases with stretching. To demonstrate their applications on soft robots, a 3D printed three-fingered soft gripper was employed with gel strips attached. During finger bending for rod grasping, the resistances of the gel strips increased, indicating stretching of the soft material. Furthermore, the strain and stress of a gel strip increased with a decrease of the rod diameter. These studies advance the application of conductive hydrogels on soft robots.

## 1. Introduction

Soft robots are a new class of robots that are primarily composed of elastic or soft materials, inspired by the softness and body compliance of biological systems [1,2,3,4]. They can continuously deform to form an infinite number of degrees of freedom to adapt to various environments. The use of compliant materials for these robots reduces their harm to the environment and to humans during interactions. These advantages broaden the application areas of traditional hard robots that are typically used in well-defined environments. Soft robots show great potential in areas such as medical rehabilitation, disaster response, special detection, and human assistance [3,5,6]. Sensing of the environment is critical for a soft robot to fulfill its outstanding performance properties such as soft biological systems, accurate manipulation, obstacle avoidance, and terrain adaption [2,7]. Studies of soft sensing materials are of great importance for the development and application of soft robots.

To accommodate the compliance and morphology of soft robots, the sensing material is required to be soft and stretchable in order to impart minimal influence on the movements of soft robots. Commercial electronic magnetic sensors were proved to be useful for tracking the positions of tagged components [8]. However, the number of sensors, as well as cost, is remarkable for monitoring the movements of soft robots with an infinite number of degrees of freedom. Optical fibers could be embedded into the bodies of soft robots to sense contact or deformation, only if without strong stretching [9]. Proprioceptive sensors based on liquid conductor (such as EGaIn) have been repeatedly reported [10,11,12,13]. The resistance of a liquid conductor, which is filling the microchannels of a soft elastomer base, changes with deformation, thereby sensing the curvature of a structure [14]. However, extreme deformations or folds in the microchannels may cause liquid disconnection or leakage. Furthermore, these sensors normally have layered structures that are inserted with microchannels, imposing great challenges for fabrication [15]. Obviously, current proprioception of soft robots relies mainly on curvature sensors. It makes sense since soft robots are actuated by generating curvatures. However, body deformations of a soft robot are often accompanied with stretching of the soft material [16,17,18], which has not been fully studied. On the other hand, contact sensing is crucial for interactions with the environment. Hence, stretch sensing and contact locating with applications to soft robots will be conducted in this work.

Conductive hydrogels (CHs) represent a class of functional materials that could sense stretching and contact with low modulus. Hydrogels are soft, stretchable, even biocompatible and transparent, with great application potential for drug delivery [19], tissue replacement [20], and wound healing [21]. CHs combine the soft-wet feature of hydrogels with electrical properties based on the ions dissolved in it [22,23]. Sensors and even actuators have been created with CHs [24,25,26]. Masarapu et al. studied the effects of pressure on the capacitance of ionic gel [23]. Ionic conductors based on polyacrylamide (PAAm) hydrogels containing lithium chloride (LiCl) salts have been used to develop touch panels. Kim et al. proposed a highly stretchable and transparent ionic touch panel that could sense contact and even touch location, and was demonstrated by writing words, playing a piano, and playing games [27]. However, the touch location was nonlinear with low locating precision, limiting its further application. Gel materials are also known for their outstanding adjustable adhesive and force-matching properties [28]. These properties would avoid sliding between a sensing material and a robot surface, which could induce sensing error or even failures [29,30,31]. A gripper with ionogel-based soft actuators has been developed by our group, which could catch and place objects by tuning the voltage [32,33]. Hence, studies of gel-based sensors are of importance for both the proprioception of soft robots and also for driving–sensing integrated soft robots in the future.

Although it has been repeatedly suggested, the practice of gel-based sensing for a soft robot is still required. In this work, we fabricated soft and stretchable gel material, introduced its sensing mechanisms, and developed a measurement setup. Touch locating abilities of gel panels of square and round shapes were studied and compared. After that, a touch locating system with improved locating accuracy was proposed with a polar coordinate system with the center of a round panel as the pole. By employing a 3D printed soft gripper, stretch sensing abilities of gel strips were demonstrated.

## 2. Materials and Methods

### 2.1. CHs Fabrication

The gel was built with mainly acrylamide (AAm) and lithium chloride (LiCl). An AAm monomer (14 wt. %) was dissolved in deionized water, together with the cross-linking agent *N*,*N*-methylenebisacrylamide (MBAA), thermal initiator ammonium persulfate (APS), and catalyst *N*,*N*,*N*′,*N*′-tetramethylethylenediamine (TMEDA) with the mole ratios of 0.028 mol %, 0.031 mol %, and 0.152 mol %, respectively. After that, 2 mol/L LiCl aqueous solution was added with respect to 2.17 mol/L AAm. Then, the evenly mixed solution was poured into a 3D printed photosensitive resin mold for 3 h at 50 °C before the gel was prepared. Gel panels in the shapes of a strip, a square, and a round were prepared for these studies.

### 2.2. Mechanical Stretching Setup

**Figure 1:**
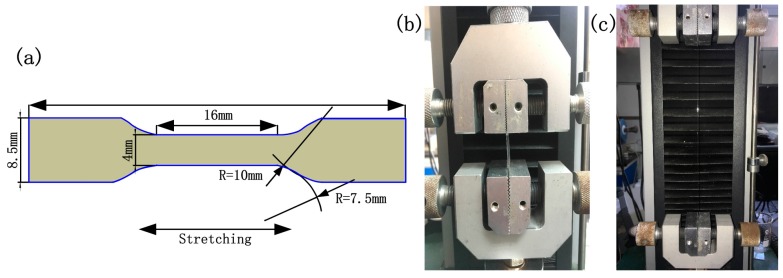
Dimensions of the test sample (**a**) and the stretching setup (**b**,**c**).

As a high polymer, the mechanical properties of the acrylamide polymer can normally be achieved through uniaxial tension and compression, or equivalent biaxial tension and compression, and shear force tests. The tensile test mainly measures the stress, strain, force, deformation, and displacement of the sample. The international standard uniaxial tension test (ISO37:2005, type 3) was adopted (Figure 1a). The samples were 2 mm in thickness. A SUNS universal testing machine (UTM 2502, SUNS, Shenzhen, China) was applied for the stretching experiment (Figure 1b,c). The relative errors of the stretching machine were ≤±0.13% and ±0.12% for the force and displacement, respectively. The stretching speed was 500 mm/min.

### 2.3. Measuring Setup

**Figure 2:**
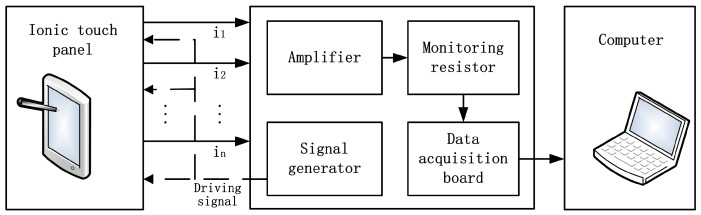
Block diagram of the measurement setup.

A block diagram of the measurement setup is shown in Figure 2. A signal generator was applied to produce a 1 kHz sinusoidal wave driving signal. The amplitude of the signal was 2 V, which was applied to each electrode. An amplifier of OPA 129 (Texas Instruments, Dallas, TX, USA) was introduced to minimize the effects of current bias.

**Figure 3:**
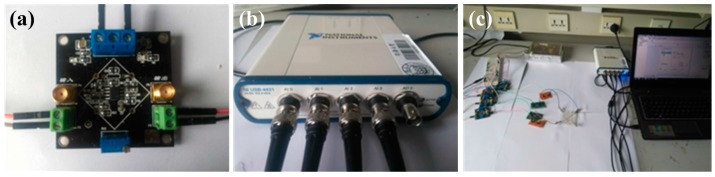
Measurement system of the touch panel. (**a**) OPA129 amplifying system; (**b**) NI-4431 four-channel data acquisition board; (**c**) The overall measurement system.

A monitoring resistor of 1 KΩ was connected in series with each corner of the panel in order to convert the current to voltage for measuring. The voltage was collected by a four-channel data acquisition board (USB-4431, National Instruments, Austin, TX, USA). The signal was then received by a computer through a custom-made LabVIEW (National Instruments, Austin, TX, USA) program for further analysis (Figure 3).

### 2.4. Sensing Mechanisms

**Figure 4:**
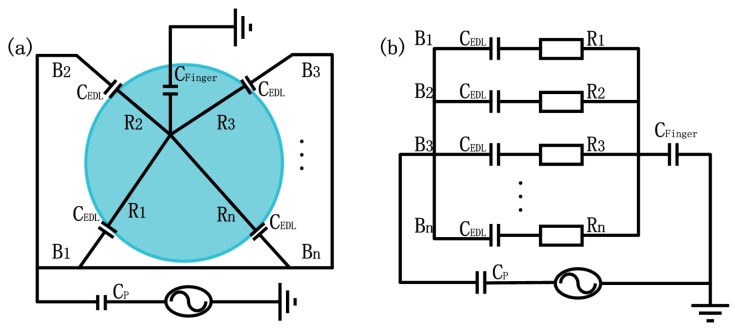
The sensing model (**a**) and electrical circuit diagram (**b**) of an ionic touch panel. $C_{\mathrm{EDL}}$ represents capacitance caused by finger touch on the panel, B1-Bn indicates sections of the panel divided by the finger touch, R and $C_{\mathrm{EDL}}$ are the resistance and capacitor of an electrical double layer for each section, and $C_{P}$ is the parasitic capacitance between the ionic gel panel and the environment.

As a surface-capacitive touch system, synchronized driving signals with the same frequency and amplitude were applied to all four corners of the panel, resulting in a uniform electrostatic field across it. When a conductor, such as a human finger, touches the panel, the touch point becomes grounded through the body, generating a potential difference between the electrode and the touch point and a current to flow from the electrode through the finger (Figure 4a). The panel is then divided into several sections (depends on the numbers of electrode) by the touch point, with a resistor (R) and a capacitor of an electrical double layer ($C_{\mathrm{EDL}}$) connecting in series for each section. All sections are connected in parallel and in series with the finger and the parasitic capacitance ($C_{P}$) between the ionic gel panel and the environment (Figure 4b).

The impedance ($Z_{i}$) of the ith path of the gel can then be written as:

(1) $$Z_{i}=R_{i}-j\frac{1}{2{\pi \mathrm{fC}}_{\mathrm{EDL}}},$$

where f is the frequency of the driving signal and $R_{i}$ is the resistance of the ith section. The capacitance per unit area of an electrical double layer is approximately 0.1 F/m^2^. The resistance of our gel panel with a size of 50 mm×50 mm×0.1 mm is about 10 kΩ. For a frequency of 10 kHz, the reactance of the double layer is

(2) $$-j\frac{1}{2{\pi \mathrm{fC}}_{\mathrm{EDL}}}\approx -1.59j.$$

Because the reactance is much smaller than the resistance of the ionic touch panel, the impedance Z=10kΩ−1.59j could be approximated by the resistance value (Z≈R). According to Figure 4b

(3) $$\frac{R_{i}}{R_{j}}=\frac{I_{j}}{I_{i}},$$

where $R_{i}$, $R_{j}$ and $I_{i}$, $I_{j}$ are the resistances and currents of the ith and jth sections, respectively. Therefore, the touch point can be located by the current relationship between the sections.

The resistance of the gel is affected by stretching. If the material is assumed incompressible ($\lambda_{x}\lambda_{y}\lambda_{z}=1$), the relationship between the resistance before (R) and after stretching ($R_{0}$) is

(4) $$\frac{R}{R_{0}}=\frac{\rho \frac{L_{i}}{S_{C}}}{\rho \frac{l_{i}}{s_{c}}}=\frac{L_{i}^{2}}{l_{i}^{2}},$$

where ρ is the resistivity, $L_{i}$, $l_{i}$ and $S_{C}$, $s_{c}$ are the lengths of interest and cross-section areas before and after stretching, respectively. It thus offers a measurement of the stretch(s) of a soft material or robot.

### 2.5. Finite Element Method Simulations

The electrical finite element method (FEM) simulation (ANSYS, Pittsburgh, PA, USA) was used to study the touch locating abilities of gel panels. The electric model was employed. Simulations were conducted using Solid 232 element. A square (100 mm × 100 mm) and a round (100 mm in diameter) gel panel were studied. A uniform electrical conductivity of 1 S/m was applied. Four electrodes with 1 V potential were fixed at each corner of the square panel or evenly distributed along the edge of the round panel. The contact was simulated by grounding a small unit (2 mm in diameter) at a designed point of a panel. Touch locations were then plotted with MATLAB (MathWorks, Neddick, MA, USA).

## 3. Results

### 3.1. Mechanical Properties

**Figure 5:**
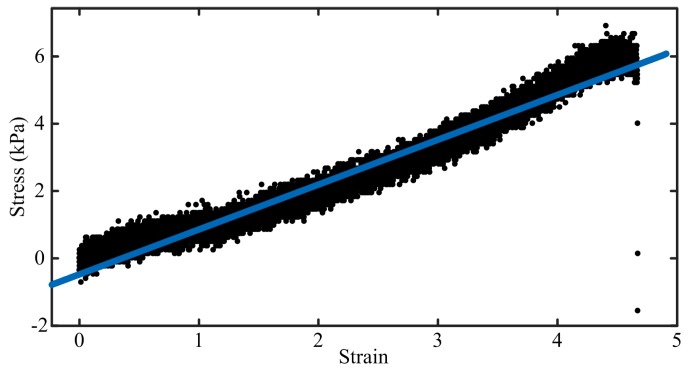
Tensile test results. *Black dots* represent individual data points; the *blue straight line* indicates the fitted line.

The strain–stress curve obtained through the uniaxial tension test is shown in Figure 5. A linear relationship between the strain (ε) and stress (σ) can be obtained as:

(5) $$\sigma =1.335\varepsilon +0.475,\;\left(R^{2}=0.960\right).$$

Therefore, the tensile elastic modulus of the gel is about 1.335 kPa; while Poisson’s ratio is 0.5 for incompressible material.

### 3.2. Touch Sensing of the Panel

**Figure 6:**
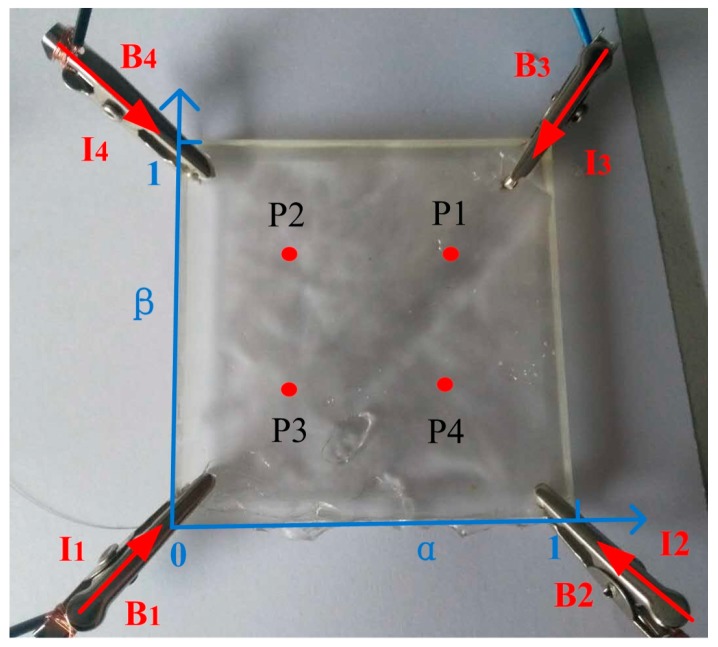
A square touch panel. Four driving signals were applied on the four corners of the panel. The panel was then divided into four sections B1–B4 with currents I1–I4 in corresponding sections. Normalized locations of four touch points P1–P4 (represented with *round dots*) could then be calculated in the coordinate system indicated by the *blue arrowed lines*.

A square panel of 50 mm×50 mm×0.1 mm was made from a 3D printed mold. Each of the four corners of the square panel was connected with a platinum electrode, which was supplied with the same driving signal. A touch by a conductor, such as a finger, divides the panel into 4 sections (B1 to B4). Normalized distances of α and β (0≤α,β≤1) were introduced to locate the touch. Four touch points and their normalized distances are shown in Figure 6.

**Figure 7:**
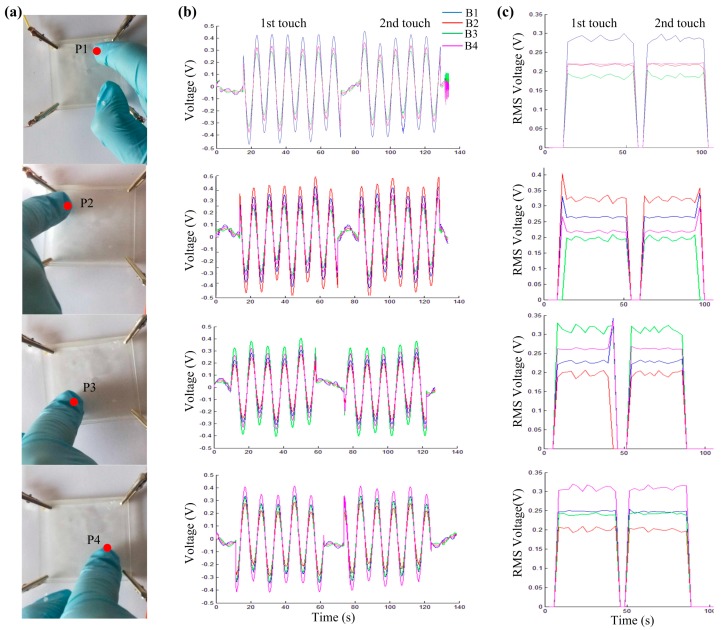
Responses of touches on a gel panel. (**a**) Photo images of the touch panel and four touch points. *Round dots* represent touch points, and P1–P4 represent touch points 1–4; (**b**) The output voltage curves of each section for two repeated touches on each touch point with a short interval between touches; (**c**) Plots of RMS values of the output voltages.

Each touch point was touched twice (Figure 7a). Each touch lasted about 60 s with a 15 s interval between touches (Figure 7b). Touches by a finger was clearly sensed by this panel. Responses of each section are shown with the root mean square values (RMS) of the voltages in Figure 7c. Voltages at these four sections responded differently at the different touch points. Locating the touch will be studied in the next section.

### 3.3. Touch Locating of the Panel

Based on Formula (3) and the sensing model in Figure 1, the normalized distance of each touch point on a touch panel connected with four electrodes (such as the touch panel shown in Figure 6) can be calculated as:

(6) $$\begin{array}{c} \alpha =\frac{\left(I_{2}{+I}_{3}\right)R}{\left(I_{1}{+I}_{2}{+I}_{3}{+I}_{4}\right)R}=\frac{V_{2}{+V}_{3}}{V_{1}{+V}_{2}{+V}_{3}{+V}_{4}},\\ \beta =\frac{\left(I_{3}{+I}_{4}\right)R}{\left(I_{1}{+I}_{2}{+I}_{3}{+I}_{4}\right)R}=\frac{V_{3}{+V}_{4}}{V_{1}{+V}_{2}{+V}_{3}{+V}_{4}}. \end{array}$$

**Figure 8:**
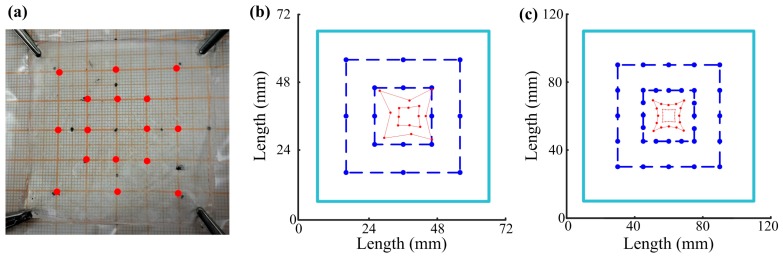
Touch locating of a square gel panel. (**a**) The touch panel with the touch locations indicated as red dots; (**b**) Sixteen points were touched on a square gel panel (60 mm × 60 mm) along two concentric squares (40 mm × 40 mm and 20 mm × 20 mm, respectively) and their locations were calculated according to Formula 9; (**c**) Thirty-two points were selected on a square gel panel (100 mm × 100 mm) along two concentric squares (60 mm × 60 mm and 30 mm × 30 mm, respectively) and their locations were simulated. *Round dots* represent individual points or locations, *dark blue dashed lines* connect touch or selected points, *red lines* connect calculated or simulated locations, and *light blue lines* indicate physical boundaries.

To verify its touch locating abilities, a square gel panel (60 mm × 60 mm) was studied by touching 16 locations along two concentric squares (40 mm × 40 mm and 20 mm × 20 mm, respectively). In order to ensure precision contacts at the designed locations, the gel panel was placed on a graph paper (mediated with a thin plastic film to avoid adhesion of the gel material on the paper) (Figure 8a). A grounded electric wire (0.2 mm in diameter) was used as a probe to contact the designed locations on the gel panel based on the graph paper in the background. The corresponding touch positions were calculated according to Formula (6) and plotted. It is clear that the touch panel showed distortion near the edges, and a stronger distortion happened to the outer square than the inner square (Figure 8b). The same results were shown by a previous study [27]. To further study the locating precision, FEM simulations were conducted; 32 touch points were evenly selected on two concentric squares (60 mm × 60 mm and 30 mm × 30 mm, respectively). Their locations were simulated. As shown in Figure 8c, the same kinds of distortion were observed for these simulated results. These distortions were due to the fact that the four resistances between a touch point and the four electrodes were not proportional to the distances between them on a square touch panel, which is obviously unfavorable to touch locating.

In order to simplify the touch locating of gel panels, two methods were considered. First, a touch panel in a different shape was considered. Then, mathematical corrections were applied to further enhance the touch locating accuracy.

**Figure 9:**
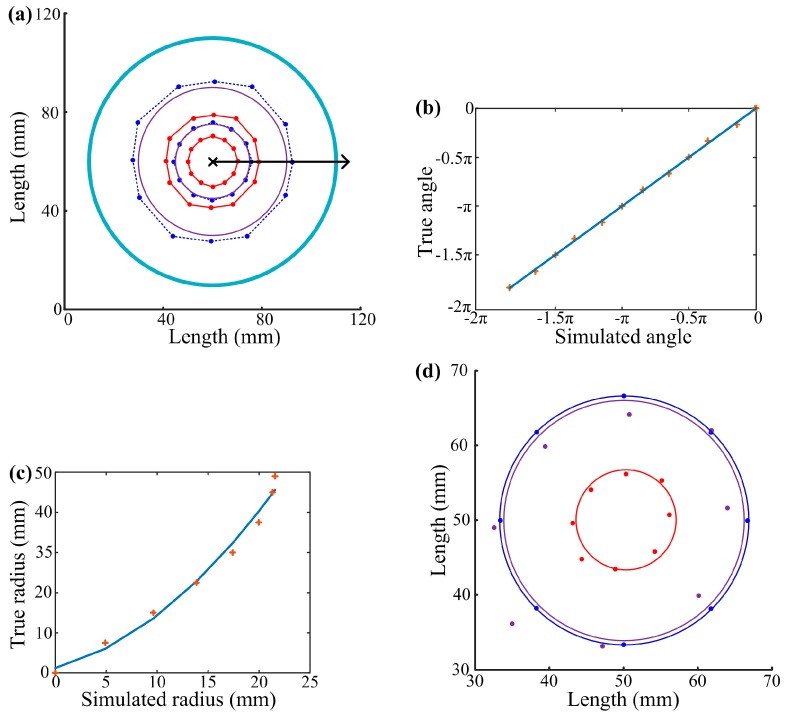
Touch locating of a round gel panel. (**a**) Twenty-four points were selected on a round gel panel (100 mm in diameter) along two concentric squares (60 mm and 30 mm in diameter, respectively) and their locations were simulated. *Round dots* represent individual points or locations, *dark blue lines* connect selected points, *red lines* connect simulated locations, *purple lines* indicate circles fitted with adjusted radii and angles according to Formulas (9) and (10), the *light blue line* indicates the physical boundary, the *cross mark* represents the pole of the polar coordinate system, and the *black line with arrow* indicates the polar axis and its direction; (**b**,**c**) Relationships between true and simulated angle and true and simulated radius of a touch point. *Cross marks* represent individual data points and *blue lines* indicate fitted mathematical relationships; (**d**) Touch locating experiments on a round touch panel. *Round dots* represent individual points or locations, the *dark blue circle* connects selected points, the *red circle* fits locations calculated with Formula (7), and the *purple circle* fits locations from Formulas (9) and (10).

A round gel panel (100 mm in diameter) was employed. Its touch locating ability was first studied by FEM simulations. Twenty-eight touch points were evenly selected on two concentric circles (60 mm and 30 mm in diameter, respectively). During these simulations, points were selected along circles rather than squares to achieve the same distance between touch points and the edge of the round touch panel, as the experiments and simulations on the square touch panel did (Figure 9a). It is obvious that the lines connecting the simulated locations keep the shapes of the lines connecting the selected points. There was no distortion for either of the circles, regardless of their distances from the panel edge. It suggests the advances of a round gel panel for touch locating. However, similar to the square touch panel, simulated locations deviated towards the center of the panel.

Considering the centrosymmetric property of circles, the panel center was chosen to be the pole of the coordinate plane. Therefore, the normalized distance of each touch point can be rewritten as:

(7) $$\begin{array}{c} \alpha =\frac{1}{2}\frac{\left(V_{2}{+V}_{3}\right)-\left(V_{1}{+V}_{4}\right)}{V_{1}{+V}_{2}{+V}_{3}{+V}_{4}},\\ \beta =\frac{1}{2}\frac{\left(V_{3}{+V}_{4}\right)-\left(V_{1}{+V}_{2}\right)}{V_{1}{+V}_{2}{+V}_{3}{+V}_{4}}. \end{array}$$

This coordinate system was further transformed to a polar coordinate system to simplify mathematical descriptions for points on a round plane. The panel center was also the pole of this new polar coordinate system; and its polar axis pointed in the same direction as the α axis of the previous coordinate system (Figure 9a). Therefore, based on Formula (7), the location of each touch point on this new polar coordinate system can be written as:

(8) $$\begin{array}{c} \rho =\sqrt{\alpha^{2}{+\beta }^{2}}=\frac{1}{\sqrt{2}}\frac{\sqrt{{\left(V_{1}-V_{3}\right)}^{2}+{\left(V_{2}-V_{4}\right)}^{2}}}{V_{1}{+V}_{2}{+V}_{3}{+V}_{4}},\\ \tan\theta =\frac{\beta }{\alpha }\frac{\left(V_{3}{+V}_{4}\right)-\left(V_{1}{+V}_{2}\right)}{\left(V_{2}{+V}_{3}\right)-\left(V_{1}{+V}_{4}\right)}. \end{array}$$

where ρ and θ are the polar radius and angle of the polar coordinate system, which could be used to describe the position of a touch point on a round gel panel.

As shown in Figure 9b, there was a linear relationship between the true and simulated polar angle of points on the panel. The relationship was

(9) $$\theta =0.994\theta^{\prime },\;\left(R^{2}=0.999\right),$$

where θ and $\theta^{\prime }$ are the true and simulated angles, respectively. The simulated and true angles were equal to each other with very small differences. It indicates that a simulated location deviated from the touch point toward the center of the panel along the opposite direction of its polar radius. How would this deviation affect the locating of touch points? In order to answer this question, 8 points that were evenly distributed on a radius from the panel center to the edge were selected and their locations were simulated. As shown in Figure 9c, the polar radius of the simulated location (simulated radius) was shorter than that of the touch point (true radius). The relationship can be written as

(10) $$r=0.06{r^{\prime }}^{2}+0.66r^{\prime }+1.19,\;\left(R^{2}=0.99\right),$$

where r and $r^{\prime }$ were the true and simulated radius, respectively. Therefore, simulated locations of touch points could be corrected by applying Formulas (7) and (8). As indicated in Figure 9a, the corrected locations showed better representation of touch points than uncorrected ones. The accuracy of this touch locating system was verified with experiments shown in Figure 9d.

### 3.4. Stretch Sensing

**Figure 10:**
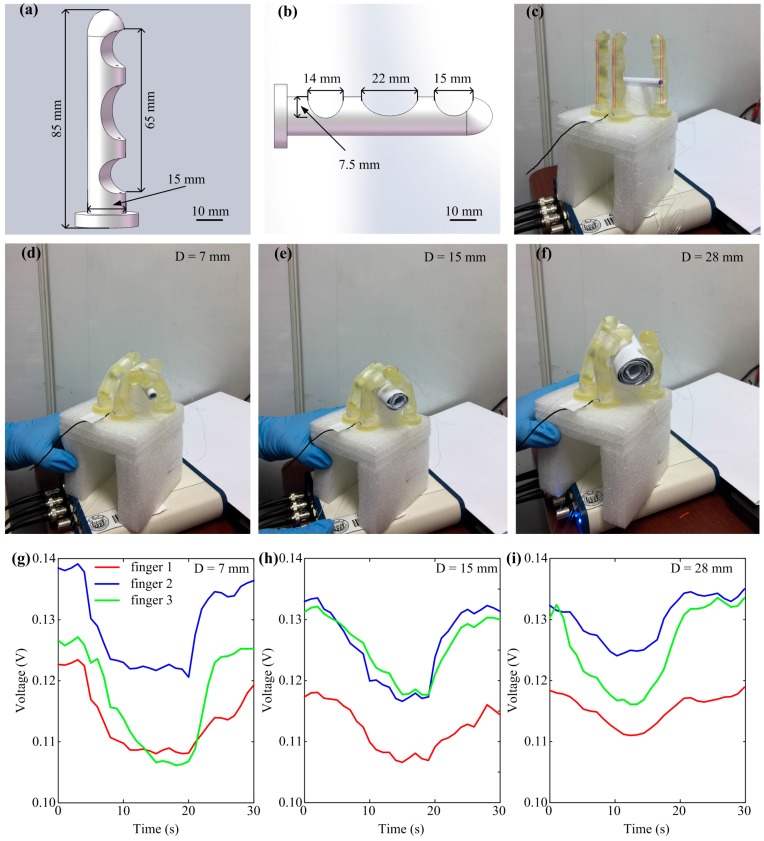
Grasp sensing of a three-finger soft gripper with gel strips. (**a**) Design of a soft finger for 3D printing; (**b**) Flexure hinges of a soft finger; (**c**) Three soft fingers of the gripper stood straight without pulley pulling and bent to grasp rods with diameters of 7 mm (**d**), 15 mm (**e**), and 28 mm (**f**); (**g**–**i**) Voltages on sampling resistors decreased when the gripper held rods with diameters of 7, 15, and 28 mm. The *red lines* in (**c**) indicate where the gel strips were attached.

Another application of gel materials to soft robots is stretch sensing. To demonstrate its stretch sensing capability, experiments were performed. The soft gripper has been a widely studied example of soft robots due to its great potential for object manipulations and safe environment interactions. Therefore, a soft gripper was developed with three fingers of the same size, based on a previous study on 3D printed soft fingers [17]. Each finger was designed with Solidworks (Dassault Systemes S.A, Concord, MA, USA) (Figure 10a) and directly printed from a rubber-like polymer (Fullcure 930 TangoPlus, Object Geometries, Rehovot, Israel) using a multimaterial printer (Eden 260V, Stratasys, Eden Prairie, Minneapolis, MN, USA). Fingers were fixed on a base to form a gripper and driven by thin threads. Finger deformations/stretches were mainly generated at the soft elliptic flexure hinges. Dimensions of these elliptic flexure hinges are shown in Figure 10b. Three gel strips (3.8 mm in diameter) were attached to the back of these elliptic flexure hinges of fingers. The resistance of each strip was measured at its two ends (65 mm in length). The tensile elastic modulus of this polymer was about 0.7 MPa [16,17,18], which was much higher than that of the gel material (1.335 kPa). Therefore, the gel strips would bend/stretch with the fingers during grasping, without affecting their own bending/stretching (Figure 10d–f). As shown by Formula (4), stretching would affect a gel strip’s resistance, as well as the voltage on the sampling resistance. It can be seen from Figure 10g–i that the voltages on the sampling resistances for each gel strip dropped while the gripper grasped the rod. Voltages dropped more with the decrease in rod diameter (from 28 mm to 15 mm to 7 mm).

The resistances of each gel strip before and after grasping were calculated; before, the average resistance of the three gel strips was achieved for each rod grasping posture. After that, the average gel length after stretching was achieved based on Formula (4). The average gel stress was obtained using Formula (6). Calculation results are reported in Table 1. A gel strip’s strain and stress increased with the decrease in the rod’s diameter for grasping, as indicated by the increase of resistance.

## 4. Discussion

Soft robots possess great environmental adaptabilities due to their soft material based bodies and possible continuous body deformations. Touch sensing and locating is crucial for environmental interactions and harm avoidance of soft robots. In this work, we studied touch locating abilities of CH-based gel panels, proposed by a previous study [27]. Both experimental and simulated results showed strong nonlinearities of touch locating on a square touch panel, confirming results of the previous study [27]. In order to improve the locating accuracy, we suggested a round touch panel with a polar coordinate system with the panel center as the pole. Locations of touch points could be calculated by the output voltages and the relationship between radii of touch points and sensed positions, since there was no angle deviation. This relationship could be obtained by panel calibration while the material stretching could be measured through the basic currents of a touch panel [27].

Touch panels with a round shape could simplify the touch locating since they are center-symmetric. This property would eliminate angle deviations between touch points and calculated positions. The locating of touch points would therefore solely depend on the radii of touch points. Further work is still required to simplify the touch locating on touch panels with a square, triangle or other arbitrary shape. This work provides a shape selecting criterion of touch panels for applications. On the other hand, a gel panel is soft and is very easy to bend or even fold to other shapes, while the proposed measurement system is still effective. This study would advance applications of gel panels in touch sensing and broaden their applications in areas such as artificial skin, soft robot, and medical care. CHs are ideal materials for the proprioception of soft robots, which is essential for their environmental interactions. Thanks to the simple structure of CHs, no complicated fabrication process is required. Their low elastic modulus ensures minimal influence on the movements of the soft robot. Its application to soft robots was demonstrated by grasping experiments with a 3D printed three-finger gripper. The movement of grasping was clearly captured by the voltage changes of the sampling resistor, which was connected in series with a gel strip attached to a finger. The voltage changes were caused by the resistance changes of the gel strip, induced by material stretching during finger bending. As shown in Table 1, the strains were small. Soft finger deformation contributed more to this gripper’s grasping than did stretching. However, material stretching affects the static and dynamic properties of a soft robot. Studies on material stretching are crucial for the control of soft robots. Pulleys driving the stretching and relaxing are basic movements of human hands and cable-driven soft hands. Gel strips offer a simple way of movement sensing. By taking finger cooperation into consideration, it would be possible to recognize grasp postures and patterns, which are of great importance to human–machine interactions, industrial hands, and medical rehabilitation of prosthetic hands.

The sensing abilities of CHs suffer from their own material properties. As discussed above, the system symmetry is important for the precise touch locating by a gel panel, including material evenness, panel shape, locations of electrode pairs, and so on. During our studies, gel panels were fabricated with molds in order to obtain controlled panel shape and thickness. However, demolding difficulties were still encountered due to its high adhesive property, which could have led to possible material unevenness across the panel surface and/or shape distortion. As shown in Figure 8a, relatively big clamps were used to connect gel panels. The positions of the electrode pairs were difficult to control precisely. In Figure 8 and Figure 9, the calculated locations of the symmetric touch points were non-symmetric, which could have been caused by the poor system symmetry. In order to avoid these problems, advanced molding/demolding techniques need to be applied. For example, molds can be made with low adhesion material (such as poly tetra fluoroethylene, or other low surface energy material) or coated with nitroglycerin or other lubricating fluid to help in demolding. Smaller electrodes would also help to improve the locating precision. Furthermore, as a high polymer, the gel material’s hysteresis curve is strongly affected by its loading. In order to obtain precise material/structure stretching with a gel strip, not only is its current deforming history needed but also its previous deforming history. As a matter of fact, materials that are used to make soft robots are normally soft and hysteresis. When attaching a soft material/structure, a gel strip’s deformation (including stretching and recovering) would be determined by that of the attached material/structure. Due to its very low stiffness, a gel strip would not affect the deformation of the measured material/structure but could sense their stretching by resistance variations.

The contact sensing of a biological system or a soft robot includes touch, pressure, friction, and so on. We have shown in this work that CHs are promising for touch locating and stretch sensing. We have studied the pressure sensing properties of gel-based parallel capacitors (publication in process). Their friction and other sensing possibilities will be studied in the future. As introduced in the introduction, some of our authors have built soft actuators based on ionogel and have proved their driving abilities both by (FEM) simulations and experimental studies [32,33]. Our further research will be carried out to develop driving–sensing integrated gel materials/structures for soft robots.

## Figures and Tables

**Table 1:** Stretching analyses of gel strips for grasping.

Parameters	Rod Diameter (mm)		
	7	15	28
**Resistance Increase (%)**	115.60	112.13	107.11
**Length Increase (%)**	107.52	105.89	103.49
**Strain (%)**	7.52	5.89	3.49
**Stress of Gel Strips (kPa)**	0.58	0.55	0.52
